# Kármán Vortex Street Driven Membrane Triboelectric Nanogenerator for Enhanced Ultra-Low Speed Wind Energy Harvesting and Active Gas Flow Sensing

**DOI:** 10.1021/acsami.2c16350

**Published:** 2022-11-02

**Authors:** Wenjian Li, Liqiang Lu, Xianpeng Fu, Chi Zhang, Katja Loos, Yutao Pei

**Affiliations:** † Department of Advanced Production Engineering, Engineering and Technology Institute Groningen, Faculty of Science and Engineering, University of Groningen, Nijenborgh 4, 9747 AGGroningen, The Netherlands; ‡ CAS Center for Excellence in Nanoscience, Beijing Key Laboratory of Micro-nano Energy and Sensor, Beijing Institute of Nanoenergy and Nanosystems, Chinese Academy of Sciences, 101400Beijing, China; § Macromolecular Chemistry and New Polymeric Materials, Zernike Institute for Advanced Materials, Faculty of Science and Engineering, University of Groningen, Nijenborgh 4, 9747 AGGroningen, The Netherlands

**Keywords:** triboelectric nanogenerator, wind energy, energy harvesting, Kármán vortex, gas leakage

## Abstract

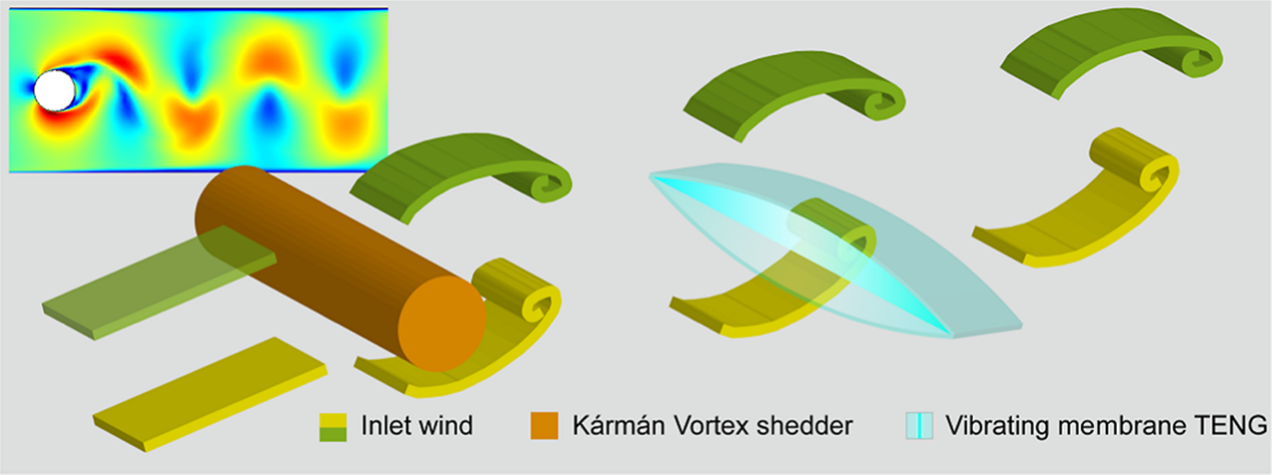

Wind energy harvesting and sensing have a huge prospect in constructing self-powered sensor nodes, but the energy transducing efficiency at low and ultra-low wind speeds is still limited. Herein, we proposed a Kármán vortex street driven membrane triboelectric nanogenerator (KVSM-TENG) for ultra-low speed wind energy harvesting and flow sensing. By introducing Kármán vortex in the KVSM-TENG, the cut-in wind speed of the KVSM-TENG decreased from 1 to 0.52 m/s that is the lowest cut-in wind speed in current TENGs. The instantaneous output density of the KVSM-TENG significantly increased by 1000 times and 2.65 times at the inlet wind speeds of 1 and 2 m/s, respectively. In addition, with the excellent energy transducing performance at the ultra-low speed range, the KVSM-TENG was successfully demonstrated to detect a weak leakage of gas pipeline (∼0.6 m/s) for alarming with high sensitivity. The interaction mechanism between the vortex and KVSM-TENG was systematically investigated. Through the simulation and experimental validation, the enhancement mechanism of vortex dependence on the cylinder diameter and placement location of KVSM-TENG was investigated in detail. The influence of parameters such as membrane length, width, thickness, and electrode gap on the performance of the KVSM-TENG was systematically studied. This work not only provided an ingenious strategy for ultra-low speed wind energy harvesting but also demonstrates the promising prospects for monitoring the air flow in the natural gas exploitation and transportation.

## 1 Introduction

The world is entering into an era of the internet of things (IoTs), big data, and artificial intelligence, where everything is expected to be connected together through numerous sensor nodes.^1^ Considering the gigantic number and spacious distribution of those senor nodes, the power supply of them remains a great challenge in the distributed IoTs.^2^ Conventional batteries can not only cause huge maintenance and replacement costs but also lead to environmental pollution when they are depleted. Therefore, self-powered sensor nodes that can meet their own power supply by harvesting energy from the ambient environment are gaining increasing attention and research interest.^3−5^ The working environment of a sensor node largely determines the harvestable energy types, such as wind, solar, vibration, human motion, water wave, and so on. For those sensor nodes placed in the wild for environmental monitoring and sensing, wind energy is one of the optimal candidates for its wide distribution and permanent presence.^6^ Wind energy harvesting is mainly realized by traditional electromagnetic generators (EMGs) in wind turbines whose cut-in wind speeds are normally over 3 m/s.^7^ However, over most of the period, the wind is in low-speed range, especially at a low altitude. It has been reported that the global average wind speed is about 3.28 m/s under the height of 10 meters,^8^ which means that the majority of wind energy in the low speed range is being wasted. In addition, the promotion and use of clean energy due to environmental problems lead to the massive growth of gas pipelines, of which the gas leakage could cause disastrous life and property loss. Hence, the monitoring and alarming of gas leakage are highly desired.

The triboelectric nanogenerator (TENG)^9,10^ based on the principle of contact electrification and electrostatic induction has demonstrated huge potential in wind energy harvesting and flow sensing.^11^ Owing to the merits of lightweight, flexibility, and excellent scalability, TENGs are exhibiting their superiority in random and low speed (2–6 m/s) wind energy harvesting compared to EMGs.^12−19^ Some TENGs with optimized structures could operate at cut-in wind speeds lower than 3 m/s, such as flow-induced vibration TENG (2.9 m/s),^17^ soft friction TENG (2.7 m/s),^18^ rolling contact electrification TENG (2 m/s),^19^ flag surface TENG (1.55 m/s),^20^ galloping TENG (1 m/s),^21^ flutter membrane-based TENG (1 m/s),^22^ and so on. To further lower the cut-in wind speed, Ren et al. proposed an ultra-stretchable TENG that could work at an ultra-low wind speed of 0.7 m/s.^23^ Lin et al. developed a pendulum-inspired TENG that had an ultrahigh mechanical triggering sensitivity.^24^ Nevertheless, despite their low cut-in wind speeds, those above-mentioned TENGs could not efficiently harvest ultra-low speed wind energy due to the low efficiency at such a low speed wind. As a result, strategies still need to be exploited and applied to boost the energy harvesting performance and efficiency of TENGs at low and ultra-low wind speeds.

Kármán vortex street,^25^ referring to continuous vortex shedding when fluids flow across blunt bodies, is always on the one hand considered as a harmful effect to infrastructures due to corresponding vortex-induced vibrations. On the other hand, it can masterly be applied for effectively energy harvesting, which has been applied in piezoelectric and EMG.^26,27^ Recently, vortex-induced vibration has also been introduced into TENGs,^28−31^ which largely improved the wind and underwater energy harvesting performance. However, the interaction between the vortex and TENGs and the enhancement mechanism are still unclear, which are important for guiding the future designs of TENGs because of their varying structures and materials. Besides, the achieved cut-in wind speeds were still not low enough for efficiently ultra-low speed wind energy harvesting and sensing.

Herein, we proposed a Kármán vortex street driven membrane TENG (KVSM-TENG) to effectively harvest wind energy, especially at ultra-low wind speeds. With a cylinder to generate the vortex, the cut-in wind speed of the KVSM-TENG decreased from 1 to 0.52 m/s. Moreover, the instantaneous output density of the KVSM-TENG dramatically increased 1000 times at the inlet wind speed of 1 m/s, increasing from ∼0.004 to ∼4 mW/m^2^. The vibration of the KVSM-TENG tended to saturate with the increase in wind speed, but the instantaneous output density still demonstrated a 2.65 times boost at the wind speed of 2 m/s. The influence of the cylinder diameter and the placement location of the KVSM-TENG in the vortex field on the output performance of the KVSM-TENG were systematically studied. Parameters of the KVSM-TENG, including membrane length, width, thickness, stretch, and electrode gap, were also investigated to realize efficiently ultra-low wind speed energy harvesting. Finally, the KVSM-TENG was demonstrated for detecting a weak leakage of gas pipeline with excellent sensitivity. This work proposed a simple, low-cost but effective device for effectively wind energy harvesting and flow sensing, especially at ultra-low wind speeds.

## 2 Results and Discussion

**Figure 1:**
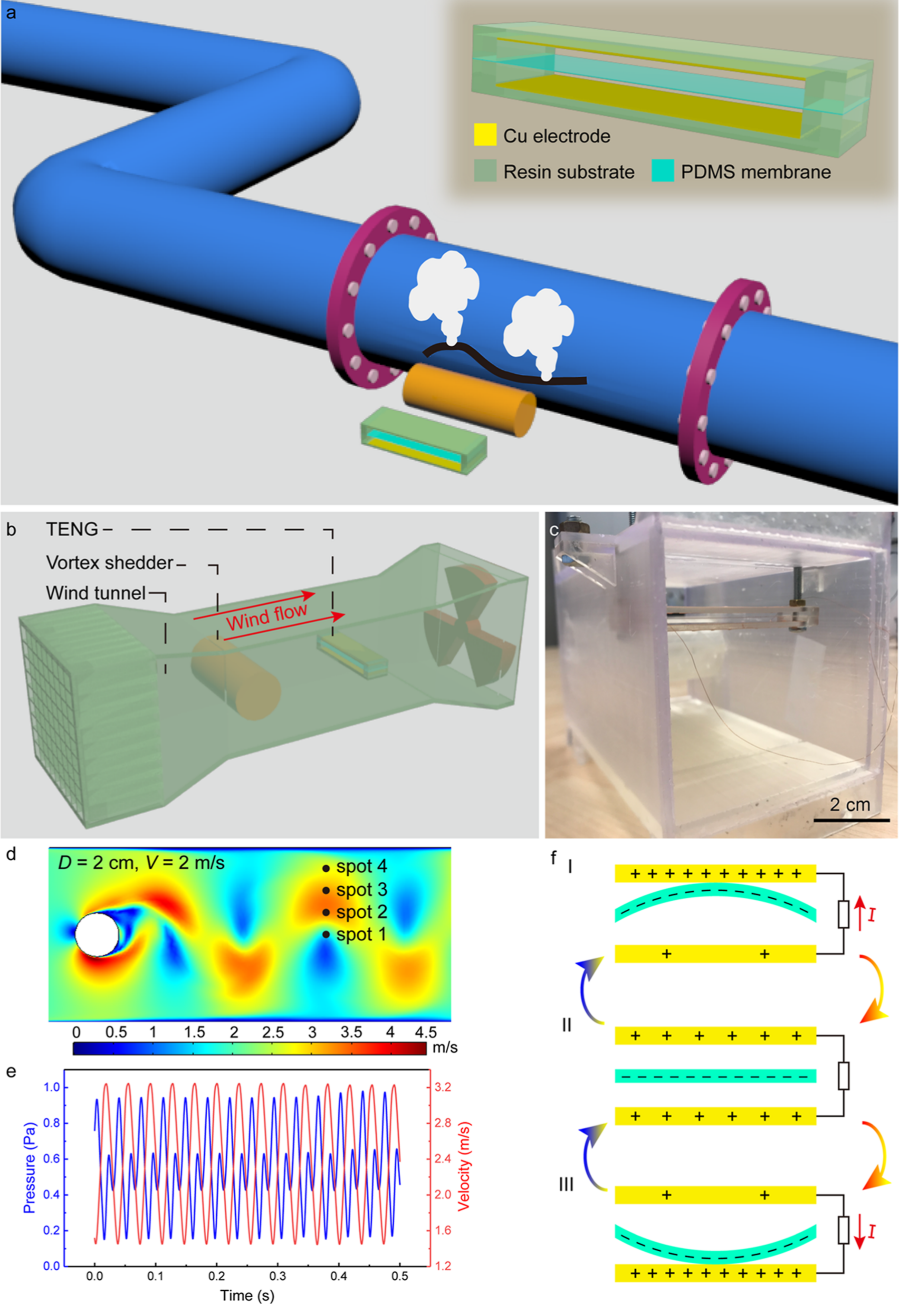
Schematic illustration of Kármán vortex street, the wind tunnel, and the KVSM-TENG. (a) Schematic illustration of using Kármán vortex street to enhance the vibration of the TENG for ultra-low wind speed energy harvesting and flow sensing. Inset: Schematic illustration of the structure of the KVSM-TENG. (b) Schematic illustration of the home-designed low speed wind tunnel. (c) Photograph of the KVSM-TENG. (d) Velocity contour of the simulated Kármán vortex street in the stationary section of the wind tunnel with a cylinder diameter of 2 cm and inlet wind speed of 2 m/s. (e) Pressure and velocity curve of the spot 3 in (d). (f) Working principle of the KVSM-TENG.

Kármán vortex street spontaneously happens when fluids (liquid or gas) flow across a blunt body from which vortices continuously shed and form a repeating pattern of swirling vortices. The velocity and pressure of the flow after the blunt body would accordingly periodically change, inducing the vibration of objects behind the blunt body and even the blunt body itself, which is usually hoped to be avoided as a harmful effect. As illustrated in Figure 1a, Kármán vortex street here was intentionally introduced to enhance the vibration of the membrane of the KVSM-TENG, aiming to boost the wind energy harvesting and flow sensing performance of the KVSM-TENG, especially at ultra-low wind speeds, such as weak gas leakage. To systematically investigate the influence of the vortex on the output performance enhancement of the KVSM-TENG, a low-speed wind tunnel was designed and homemade. As can be seen in Figure 1b, in the stationary section of the wind tunnel (KVSM-TENG) a cylinder vortex shedder was placed in the front to create Kármán vortex, after which a membrane TENG was placed parallel to the cylinder. Figure S1 shows the photos of the wind tunnel and the cylinder vortex shedder. The dimension of the stationary section as well as the placement location of the cylinder and the KVSM-TENG are illustrated in Figure S2, from which one can see that the diameter (*D*) of the cylinder and the placement height (*H*) of the KVSM-TENG in the channel can be tuned (the placement heights of 0, 1, 3, and 4 cm are hereafter referred as spot 1, spot 2, spot 3, and spot 4, respectively). The KVSM-TENG was designed in a sandwiched structure. To be more specific, an elastic polydimethylsiloxane (PDMS) membrane was sandwiched with a certain gap between two copper electrodes that were attached on two 3D printed U-shaped resin substrates, as demonstrated in the inset in Figure 1a. Then, the KVSM-TENG was tightly assembled at the two ends by long screws that were also used to adjust the placement height of the KVSM-TENG in the wind tunnel (Figure 1c).

The vortex street forms only when the Reynolds (*Re*) number of the fluid is in a certain range. For a cylinder vortex shedder, 47 < *Re* < 10^5^ is required for the formation of vortex street. The *Re* number is defined by the following formula
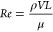
1where ρ is the density of the fluid (kg/m^3^), *V* is the flow speed (m/s), *L* is the characteristic linear dimension (m), and μ is the dynamic viscosity of the fluid (m^2^/s). Here, the characteristic linear dimension is the diameter of the cylinder.

The frequency of the generated vortex street in a finite channel^32^ can be calculated by
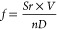
2where *D* is the diameter of the cylinder, and *n* is the ratio of the effective flow width to the total width of the channel. *Sr* is the dimensionless Strouhal number, which can be calculated using the following empirical formula
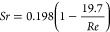
3

To verify the formation of vortex street in the home-designed wind tunnel, a simulation was conducted using finite element analysis in Comsol Multiphysics 5.2a, where different cylinder diameters (1, 2, 3, 4, and 5 cm) and inlet wind speeds (0.5, 1, 2, 3, 4, and 5 m/s) were applied. Figure S3 shows the simulated pressure and velocity contours when the *Re* number is the minimum (321.5, *D* = 1 cm, *V* = 0.5 m/s) and the maximum (16,078.2, *D* = 5 cm, *V* = 5 m/s), respectively, from which one can clearly see that vortex street can be generated even at the ultra-low wind speed range. Figure S4 demonstrates the fast Fourier transform (FFT) frequency analysis results of the simulated pressure with different cylinder diameters, while the inlet wind speed increased stepwise from 0.5 to 5 m/s. It can be seen that the frequencies of the simulated vortex streets fit well with the frequencies calculated via eq 2, revealing that controllable vortices can be created in the home-designed wind tunnel. The vortex street can result in periodic changes in the pressure and velocity fields after the cylinder, causing the vibration enhancement of the KVSM-TENG. As an example, Figure 1d shows the velocity contour when *D* = 2 cm and *V* = 2 m/s, and the pressure and velocity profiles of the spot 3 are shown in Figure 1e, which clearly demonstrates the periodic changes of the pressure and velocity. The location of the KVSM-TENG behind the cylinder is directly related to the strength of vortex street, so the velocity and pressure of the spots at different characteristic lengths (CLs) after the cylinder is first investigated through the simulation. As can be seen from Figure S5, the pressure difference of the spots first increases and reaches the maximum at 3 CL and then decreases, while the velocity difference decreases with the distance increases. Therefore, the KVSM-TENG is placed 2.5 CLs away from the cylinder. When the wind flow comes, the PDMS membrane of the KVSM-TENG tends to vibrate and touch the two copper electrodes. After several up-down reciprocating vibrations, negative charges accumulate on the surface of the PDMS, while equal amount of positive charges accumulate on the two copper electrodes, as a result of the contact electrification effect as depicted in state II in Figure 1f. When the PDMS membrane vibrates upward and touches the upper electrode, electrons will flow from the upper electrode to the bottom electrode due to the electrostatic induction effect (state I). Similarly, electrons will flow from the bottom electrode to the upper electrode when the PDMS membrane touches the bottom electrode (state III). Excited by the vortex street, the vibration of the PDMS membrane tends to be enhanced and thus increasing the contact area between the PDMS membrane and electrodes. In this way, the output performance of the KVSM-TENG can be boosted, especially in the ultra-low wind speed range.

**Figure 2:**
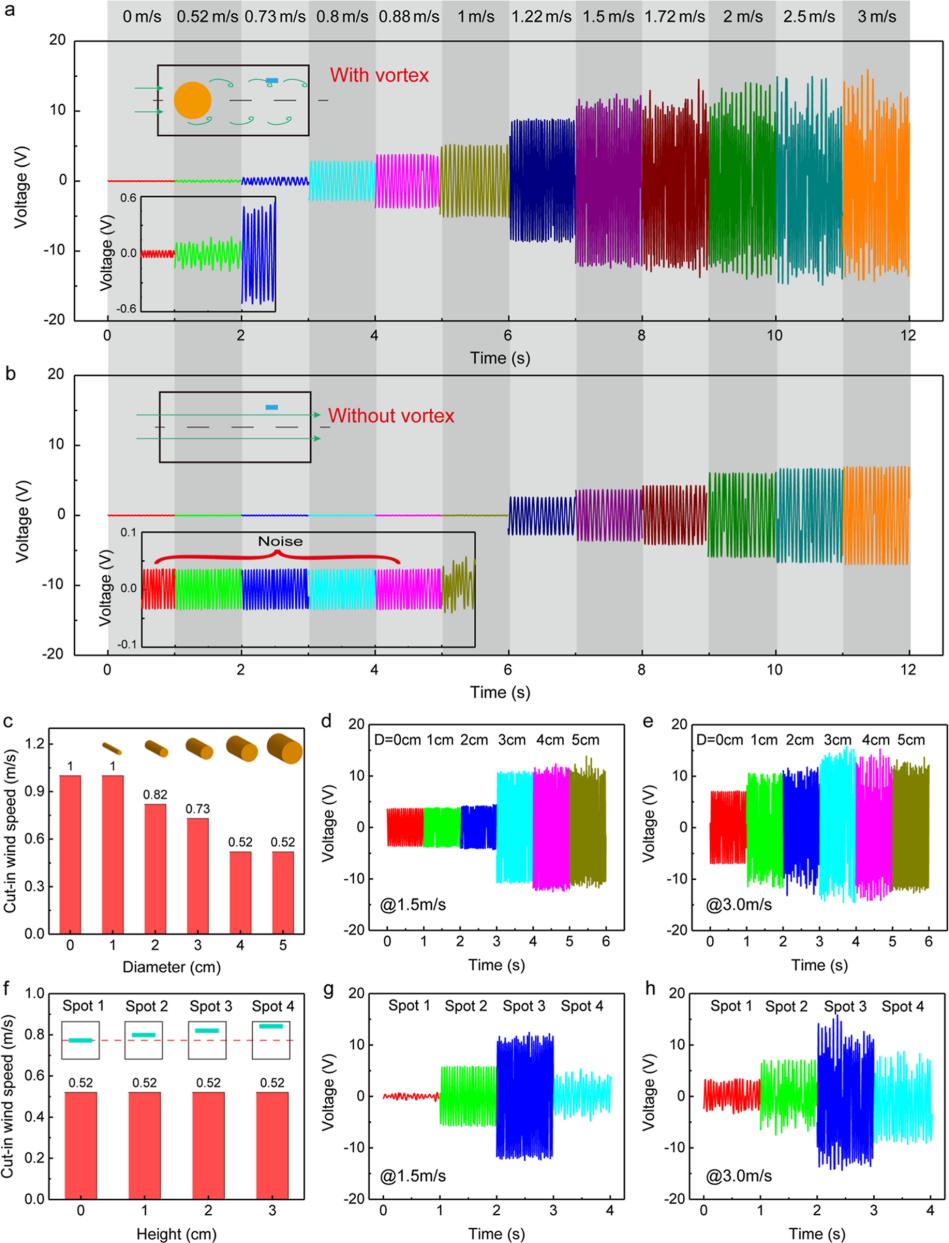
Characterization of the influence of the vortex street on the output of the KVSM-TENG. The output open-circuit voltage of the KVSM-TENG with (a) and without (b) the excitation of the vortex street (*D* = 4 cm, *H* = 2 cm). (c) Cut-in wind speeds of the KVSM-TENG under different cylinder diameters. The outputs of the KVSM-TENG under different cylinder diameters at the wind speeds of 1.5 (d) and 3 m/s (e), respectively. (f) Cut-in wind speeds of the KVSM-TENG located at different spots. The outputs of the KVSM-TENG at different spots at the wind speed of 1.5 (g) and 3 m/s (h), respectively.

Figure 2a,b shows the output open-circuit voltage of the KVSM-TENG under different inlet wind speeds with and without the excitation of the vortex, respectively. Here, the cylinder diameter was 4 cm and the KVSM-TENG was placed at spot 3. The influence of different cylinder diameters and placement spots on the output will discussed in detail later. As can be seen in Video S1, with the vortex street, the KVSM-TENG began to have a clear output voltage at an ultra-low wind speed of 0.52 m/s that was the lowest stable wind speed realized by the homemade wind tunnel, while the KVSM-TENG could only generate a tiny output voltage at 1 m/s if without the vortex. It means that the cut-in wind speed of the KVSM-TENG is dramatically decreased from 1 to 0.52 m/s. Moreover, the output voltage of the KVSM-TENG is largely enhanced over a wide range of wind speed (0.52–3 m/s), for example, increased from 0.1 to 10 V at 1 m/s (100 times), from 12 to 27 V at 2 m/s (2.25 times), and from 14 to 28 V at 3 m/s (2 times). The output short-circuit current and transferred charges of the KVSM-TENG demonstrated the same enhancement effect, as shown in Figures S6 and S7, respectively. Obviously, the output of the KVSM-TENG was greatly boosted by introducing the Kármán vortex street, especially at the ultra-low wind speed range. The first reason is that the high-frequency periodic changes of the pressure and velocity in the flow field can enhance the vibration of the PDMS membrane (Videos S2 and S3). The second reason is the average velocity of the wind flow increased after vortex shedding, as can be seen in the simulation result (Figure 1e) and experimental wind calibration result (Figure S8). The vibration frequency of the membrane with and without the excitation of vortex is also analyzed, as demonstrated in Figure S9. Without the vortex street, the vibration frequency of the membrane increased linearly and slowly with the increase in the inlet wind speed from 1 to 3 m/s. After adding the cylinder, the vibration frequency of the membrane first increased slowly and linearly when the wind speed was increased from 0.52 to 1 m/s. The vibration frequency almost doubled when the wind speed was 1.22 m/s and then decreased slightly when the wind speed was further increased from 1.22 to 3 m/s. The results revealed that the vortex mainly enhanced the vibration amplitude of the membrane at ultra-low wind speed (<1 m/s). When the wind speed was further increased, the generated vortex was strong enough to change the vibration mode of the membrane and thus dramatically increased the vibration frequency and amplitude. The reason why the vibration frequency decreased slightly is attributed to the vacuum adsorption effect, which will be discussed later.

The cylinder diameter has a critical influence on the intensity (frequency, pressure and velocity) of the vortex, thus affecting the enhancement of the output performance of the KVSM-TENG. First, simulations with different cylinder diameters were conducted to study the corresponding pressures and velocities in the vortex flow field. The monitored pressure and velocity of spot 1 with different cylinder diameters at different inlet wind speeds (0.5, 1, 2, and 3 m/s) are demonstrated in Figure S10. As can be seen from the results, both the pressure and the velocity increased when the cylinder diameter increased from 1 to 5 cm. When the diameter was 5 cm, the pressure and velocity largely increased but became more chaotic compared to the other cases using smaller cylinders. The output of the KVSM-TENG was then tested under different cylinder diameters. Figure 2c shows the cut-in wind speeds of the KVSM-TENG under different cylinder diameters. The cut-in wind speed decreased when the diameter increased. Here, the outputs only under the inlet speed of 1.5 and 3 m/s were extracted for comparison and discussion. At the inlet speed of 1.5 m/s, the output of the KVSM-TENG increased slowly until the diameter increased to 2 cm, and then sharply increased when the diameter was 3 cm and finally almost saturated when the diameter kept increasing, as shown in Figure 2d. It is worth nothing that the output of the KVSM-TENG slightly decreased when the diameter increased from 3 to 5 cm at the wind speed of 3 m/s, as depicted in Figure 2e. This is because when the vibration of the PDMS membrane is stronger, the impact force will be very large between the membrane and the electrode, so that the air is expelled to form a vacuum adsorption in the contact area. Due to the vacuum adsorption effect, a small part of the PDMS membrane is stuck on the electrode, as shown in Figure S11; thus, the vibration amplitude of the membrane decreases. However, it is worth noting that the vacuum adsorption effect can be prevented by surface micropatterning of the triboelectric surfaces to decrease the surface stickiness while increasing the output performance simultaneously. Therefore, the cylinder diameter is determined as 4 cm for the following investigations.

The placement location of the KVSM-TENG in the vortex field is another important factor that determines the best vibration enhancement of the KVSM-TENG. The pressure and velocity of the vortex at different heights (spot 1, spot 2, spot 3, and spot 4) and different inlet wind speeds were first studied through simulation, and the results are shown in Figure S12. As can be seen, spot 3 has the maximum peak–peak pressure and velocity in most conditions. The experimental test results of the output of the KVSM-TENG located at the different heights are shown in Figure S13, which demonstrates that the output of the KVSM-TENG at spot 3 had a significant enhancement, while the outputs at spot 1 and spot 2 were even smaller than that of without vortex showed above. This interesting phenomenon can be attributed to the structure of the KVSM-TENG in which the resin substrates can act as wind shelter at low spots. As can be seen from the streamlines inside the channel (Figure S14a), the wind flow vibrates at a large amplitude near the center of the channel (spots 1 and 2), while that near spot 3 natures a swing with moderate amplitude. As a result, the substrates of the KVSM-TENG at spots 1 and 2 can act as shelters that block the wind interacting with the membrane. However, the moderate vibration wind near spot 3 can easily flow through the gap between the two substrates and resonant with the membrane (Figure S14b). The output at spot 4 was similar with that without vortex because the vibration of the wind flow caused by vortices is almost negligible near the wall of the flow channel. However, an ultra-low cut-in wind speed of 0.52 m/s was achieved at all the spots, as summarized in Figure 2f, which reveals that the vortex has an important role in lowering the cut-in wind speed of the KVSM-TENG. Figure 2g,h are the summary of the outputs of the KVSM-TENG at wind speeds of 1.5 and 3 m/s, respectively.

**Figure 3:**
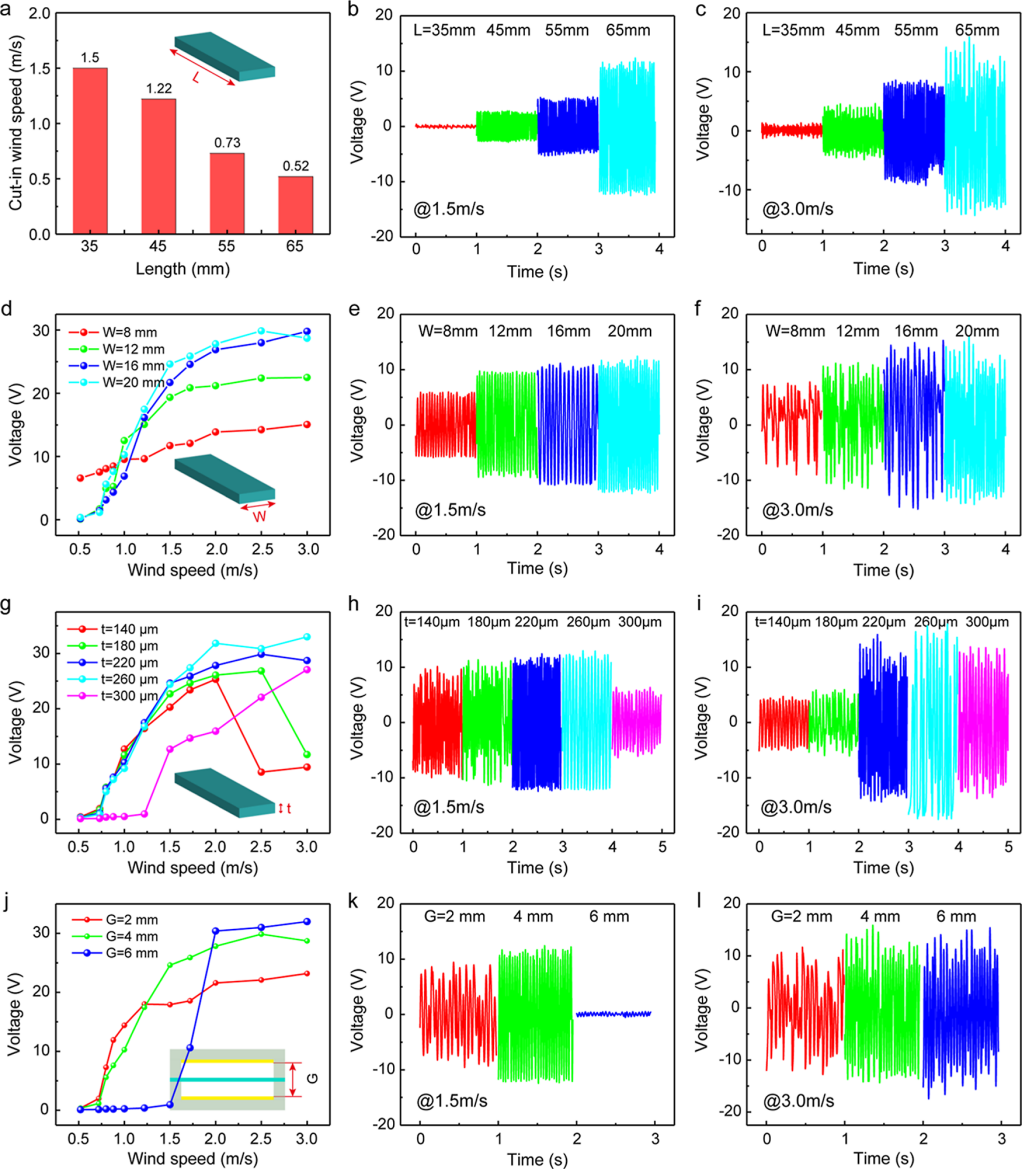
Influence of membrane length, width, thickness, and electrode gap on the output of the KVSM-TENG. (a) Cut-in wind speeds of the KVSM-TENG with different membrane lengths. The output voltage of the KVSM-TENG with different membrane lengths at the wind speeds of 1.5 (b) and 3 m/s (c) (*W* = 20 mm, *t* = 220 μm, *G* = 4 mm). (d) The output voltage of the KVSM-TENG with different membrane widths. The outputs of the KVSM-TENG with different membrane widths at the wind speeds of 1.5 (e) and 3 m/s (f) (*L* = 65 mm, *t* = 220 μm, *G* = 4 mm). (g) The output voltage of the KVSM-TENG with different membrane thicknesses. The outputs of the KVSM-TENG with different membrane thicknesses at the wind speeds of 1.5 (h) and 3 m/s (i) (*L* = 65 mm, *W* = 20 mm, *G* = 4 mm). (j) The output voltage of the KVSM-TENG with different gaps between two electrodes. The outputs of the KVSM-TENG with different gaps at the wind speeds of 1.5 (k) and 3 m/s (l) (*L* = 65 mm, *W* = 20 mm, *t* = 220 μm), respectively.

The structural parameters of the KVSM-TENG can definitely affect the interaction/vibration of the membrane with the vortex street. The influence of the structural parameters, including membrane length, width, thickness, stretch, and electrode gap, on the output of the KVSM-TENG was systematically investigated. All the subsequent experiments were conducted with the cylinder diameter of 4 cm and placement location of spot 3. Figure 3a shows the cut-in wind speeds of the KVSM-TENG with different membrane lengths (35, 45, 55, and 65 mm). The longer the membrane was, the easier the membrane vibrated, so the lower the cut-in wind speed was. With increased membrane length, the contact area between the membrane and the electrodes also increased, leading to the increase in the output of the KVSM-TENG, as can be seen from Figure 3b,c. Figure 3d demonstrates the outputs of the KVSM-TENG with different membrane widths (8, 12, 16, and 20 mm; and the membrane length is determined as 65 mm). When its width was 8 mm, the membrane was more inclined to vibrate, so the KVSM-TENG could have a high output even at the ultra-low wind speed of 0.52 m/s. However, it is also easier for it to meet the vacuum adsorption problem at higher wind speed, which can be found from the irregular outputs as shown in Figure 3f. When the inlet wind speed is beyond 1 m/s, the KVSM-TENG would have considerable vibration regardless of the membrane width, so the output of the KVSM-TENG depends more on the effective contact area, resulting in an increase of the output with the increase in the width of the membrane (Figure 3e). Similarly, the membrane with smaller thickness tended to vibrate easier and meet the vacuum adsorption problem earlier, as can be seen from the significant drops in the outputs of the KVSM-TENG with thicknesses of 140 and 180 μm, respectively (Figure 3g,i). The KVSM-TENG demonstrated a similar trend between the thickness of 220 and 260 μm, while the membrane was already stiff to vibrate at the thickness of 300 μm. The outputs of the KVSM-TENG with the thicknesses of 140 and 180 μm were smaller than those of 220 and 260 μm thick membrane at 1.5 m/s (Figure 3h), which is attributed to the appeared slight vacuum adsorption effect as can be seen from their irregular outputs. A small stretch of the membrane can largely affect its vibration; hence, the output of the KVSM-TENG under different stretches (−0.5, 0, 0.5, and 1 mm) was tested, as shown in Figure S15. When the membrane was stretched, the vibration is hardly generated in ultra-low wind speed range, while a stronger vibration can be excited in higher wind speed range. Interestingly, when the membrane was slightly released (−0.5 mm) into a suspending state, the vacuum adsorption effect happened first at the wind speed of 1.5 m/s and then disappeared and finally appeared again. The reason is that the membrane in the suspending state has a larger contact area with the electrodes and smaller elastic resilience at low wind speed and thus more easy to be stuck on the electrodes. When the wind speed increases, the vibration of the membrane is enhanced, resulting in the larger elastic resilience to overcome the vacuum adsorption effect. The influence of the gap between the two electrodes was also investigated, and the results are presented in Figure 3j–l. With a smaller gap, it is easier for the membrane to contact the electrodes, resulting in higher output at ultra-low wind speeds but also earlier to have the vacuum adsorption effect. When the gap is large enough (6 mm), the vacuum adsorption can hardly appear, but the output of the KVSM-TENG was small at low speeds.

**Figure 4:**
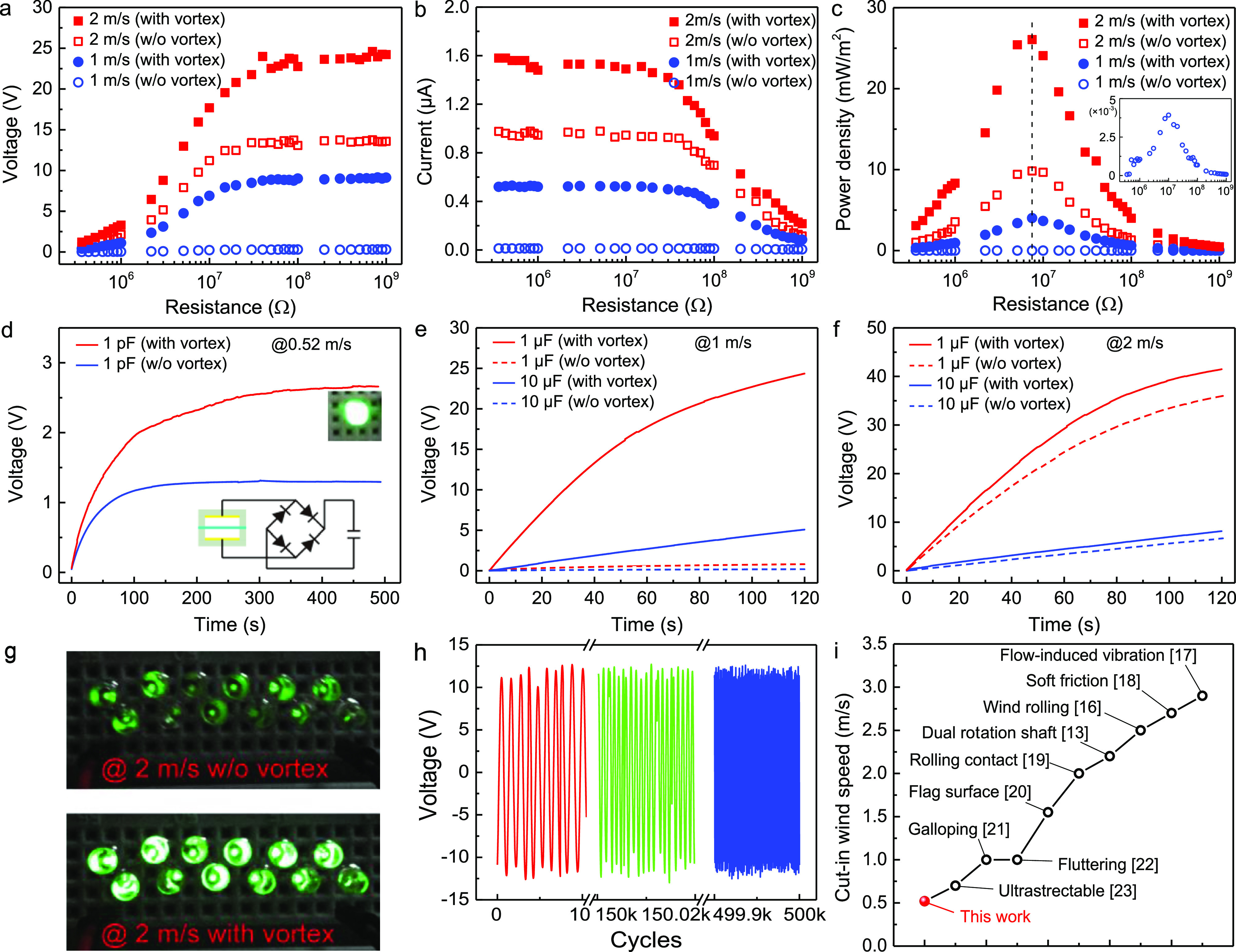
Output comparison of the KVSM-TENG with and without the vortex street. (a) Output voltage, (b) current, and (c) power density of the KVSM-TENG under external loads with and without the vortex street. (d) Charging a 1 pF capacitor at the ultra-low wind speed of 0.52 m/s. Charging a 1 and 10 μF capacitor at the wind speed of 1 (e) and 2 m/s (f), respectively. (g) Lightning 12 LEDs at the wind speed of 2 m/s. (h) Stability test of the KVSM-TENG over 500,000 cycles. (i) Comparison of the cut-in wind speeds of representative TENG works for low-speed wind/breeze energy harvesting.

With the optimized parameters, including cylinder diameter (4 cm), location (spot 3), membrane length (65 mm), width (20 mm), thickness (220 μm), stretch (0 mm), and electrode gap (4 mm), the power density and capacitor charging ability of the KVSM-TENG with and without the vortex street were compared. Impedance matching of the KVSM-TENG was tested under different external resistance to calculate the maximum instantaneous output power density. Figure 4a shows the output voltage of the KVSM-TENG under external loads with and without the vortex street at the wind speed of 1 and 2 m/s, and Figure 4b presents the output current. The output voltage first increased with the increase of the external load and finally saturated at the open-circuit voltage, while the output current demonstrated a reverse trend, decreasing from the short-circuit current. As a consequence, the instantaneous output power density of the KVSM-TENG first increased and reached the maximum at the load of 7.5 MΩ and then decreased, as shown in Figure 4c. It is clear to see that the instantaneous output power density of the KVSM-TENG was greatly boosted with the excitation of the vortex street. Specifically, the power density was initially 0.004 and 9.8 mW/m^2^ without introducing the vortex street at the wind speeds of 1 and 2 m/s, respectively, and greatly increased to 4 and 26 mW/m^2^ with the excitation of the vortex street at the corresponding wind speeds. This means that the amplification factor of the vortex to the instantaneous output power density was 1000 and 2.65 at the wind speeds of 1 and 2 m/s, respectively. The average powers of the KVSM-TENG under the matched impedance (7.5 MΩ) at the wind speeds of 1 and 2 m/s with and without the vortex were also measured at 0.53 versus 0.005 and 3.72 versus 1.46 μW, respectively (Figure S16). The reason why the amplification factor decreased with the increase in wind speed was mainly attributed to the fact that the vibration of the membrane tended to reach a saturation at high wind speeds.

The energy harvested by TENGs is expected to be stored in batteries or capacitors for powering sensor nodes. The charging ability of the KVSM-TENG with and without the vortex street was characterized by charging different capacitors (1 and 10 μF). The energy harvesting capability of the KVSM-TENG at the ultra-low wind speed of 0.52 m/s was first verified by charging a 1 pF capacitor using a rectifier bridge. As can be seen from Figure 4d, with the vortex, the KVSM-TENG could keep charging the capacitor to 2.7 V in 500 s and finally light a LED, while the voltage of the capacitor without the vortex could only rise up to 1.3 V as a result of electromagnetic noise. Then, the capacitor charging ability was compared by charging capacitors at the wind speeds of 1 and 2 m/s, respectively. Taking the capacitor of 1 μF as an example, the KVSM-TENG with the vortex could charge the capacitor to 24.4 V in 120 s at the wind speed of 1 m/s, while that without vortex could only reach 0.82 V, showing an enhancement of 30 times (Figure 4e). At the wind speed of 2 m/s, the enhancement became weaker with a voltage of the capacitor (1 μF) of 41.5 V (with vortex) and 36 V (without vortex) in 120 s, that is, a 1.15 times enhancement. Large capacitors can also be quickly charged to the required voltage to power daily electronics, and Figure S17 shows the charging process of a 47 μF capacitor. 12 LEDs can be powered by the KVSM-TENG at the wind speed of 2 m/s, and the brightness of the LEDs under vortex excitation is much higher than that without the vortex (Figure 4g). The stability of the KVSM-TENG was not affected at all even though the membrane vibration was much stronger under the excitation of the vortex. As demonstrated in Figure 4h, the output of the KVSM-TENG with the vortex was quite stable over 500,000 cycles, revealing that the influence of the vortex on the vibration mode of the membrane was stable and sustainable. Here, the wind speed was set as 1.5 m/s because of the obvious enhancement caused by the vortex at this speed, while the vibration was not reached the saturation.

It is worth noting that the cut-in wind speed achieved in this work by introducing the Kármán vortex street was almost the lowest one reported among all wind energy harvesting TENGs. A comparison of the cut-in wind speeds of some representative TENG works aimed for low-speed wind/breeze energy harvesting is presented in Figure 4i.^13,16−23^ The importance is, unlike through the complicated TENG design in other works, the method is extremely simple in this work by placing a vortex shedder in front the TENG to generate the Kármán vortex street, and this method has the potential to be extended to any types of TENG for wind energy harvesting. A promising and feasible way to further lower the cut-in wind speed and enhance energy harvesting performance in future work is combining TENG structure optimization and vortex street. Compared to other wind energy harvesting mechanisms (e.g., electromagnetic and piezoelectric) using vortex street, as listed in Table S1, the cut-in wind speed of electromagnetic and piezoelectric devices are usually high and have a small output voltage. On the contrary, this work based on triboelectricity demonstrated an ultra-low cut-in wind speed as well as a high output voltage, which reveals the huge potential in ultra-low wind speed energy harvesting and wind flow sensing.

**Figure 5:**
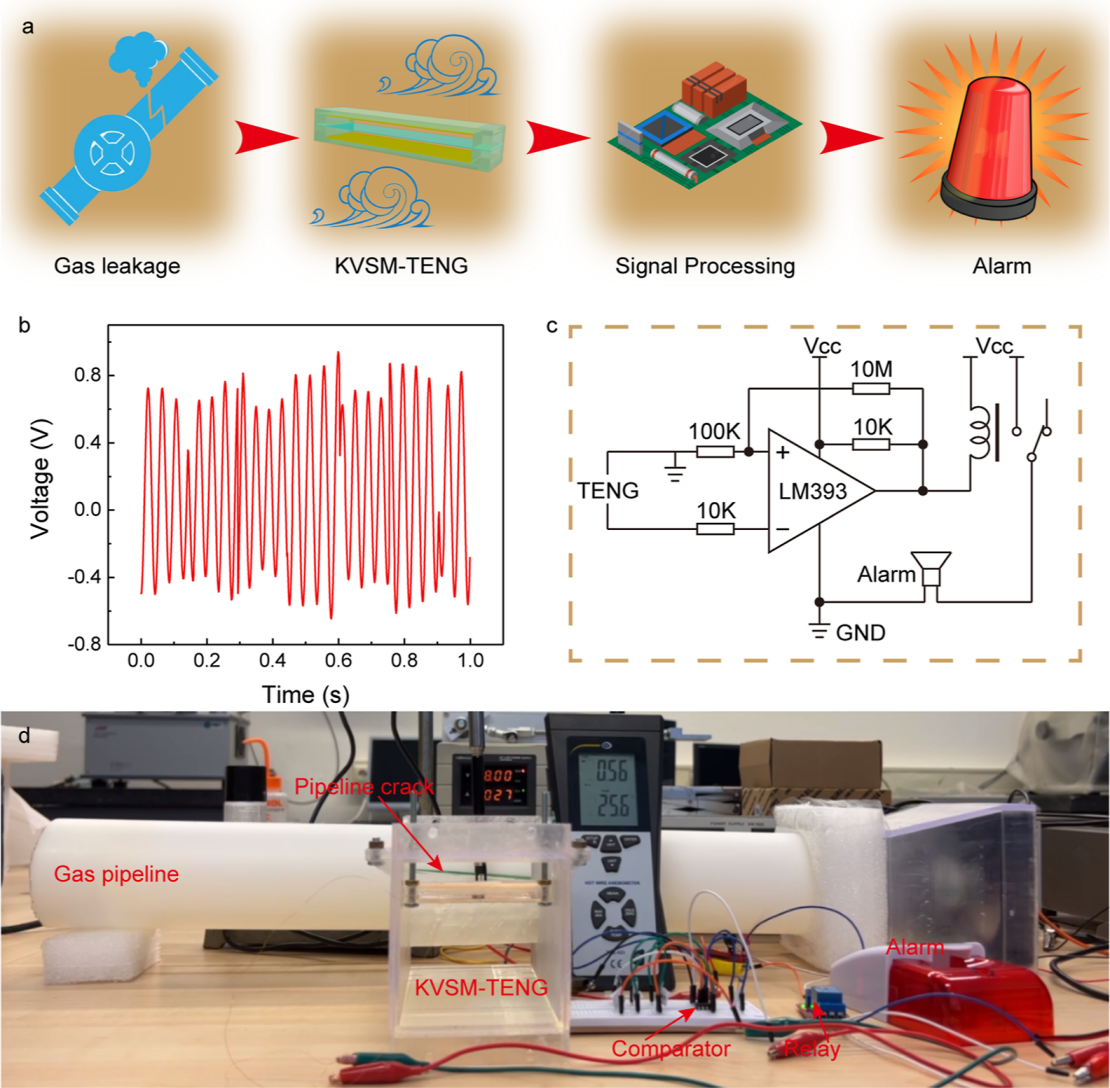
Weak gas leakage alarming based on the KVSM-TENG. (a) System block diagram of the gas leakage alarming system. (b) Voltage output of the KVSM-TENG under a weak gas leakage. (c) The signal processing circuit of the gas leakage alarming system. (d) The setup of the gas leakage alarming application.

To further reveal the potential of the proposed KVSM-TENG in ultra-low speed wind energy harvesting and flow sensing, a weak gas leakage alarming application was successfully demonstrated. The KVSM-TENG was placed in front of the crack of a handcrafted gas pipeline, as illustrated in Figure 1a. When there is a gas leakage of the pipeline, the KVSM-TENG can be triggered by the weak wind flow. Then, a comparator converts the low-energy sinusoidal-like signal from the KVSM-TENG to the TTL level to control the alarm through a relay, as depicted in Figure 5a. Figure 5b and Video S4 show the output of the KVSM-TENG under a weak gas leakage with a wind speed at ∼0.6 m/s. The signal processing circuit from the KVSM-TENG to the alarm is shown in Figure 5c. The setup of the gas leakage alarming application is demonstrated in Figure 5d. As can be seen, the flow speed of the leaked gas was about 0.56 m/s (which dynamically changing from 0.5 to 0.6 m/s). Video S5 demonstrates the sensitive real-time alarming for the weak gas leakage by using the KVSM-TENG.

## 3 Conclusions

In conclusion, we demonstrated a KVSM-TENG for wind energy harvesting and flow sensing, especially at the ultra-low wind speed. Simulations and experiments were conducted to investigate the influence of the size of the cylinder shedder and TENG’s placement location on the output enhancement. The influence of the structural parameters of the KVSM-TENG such as membrane length, width, thickness, and electrode gap were systematically studied. With the optimized parameters, the cut-in wind speed of the KVSM-TENG was significantly decreased from 1 to 0.52 m/s with the excitation of the vortex. Moreover, the instantaneous output power density of the KVSM-TENG greatly increased by 1000 times and 2.65 times at the inlet wind speeds of 1 and 2 m/s, respectively. Accordingly, the charging speed of the KVSM-TENG when charging a capacitor of 1 μF was enhanced by 30 times and 1.15 times at the wind speed of 1 and 2 m/s, respectively. What is more, a weak gas leakage alarming application was successfully demonstrated to further reveal the potential of the proposed KVSM-TENG in ultra-low speed wind energy harvesting and flow sensing.

## 4 Experimental Section

### 4.1 Fabrication of the KVSM-TENG

PDMS was prepared by mixing the base and curing agent in a weight ratio of 10:1 followed by degassing in a vacuum chamber until the bubbles disappeared. 2.5 g of PDMS mixture was slowly dripped on a 4 inch silicon wafer during the process of spin coating at 1000 rpm with different spin times for achieving different thicknesses (30, 45, 50, 60, and 80 s for 300, 260, 220, 180, and 140 μm, respectively), after which the silicon wafer coated with the PDMS mixture was cured in an oven at 80 °C for 2 h. PDMS membranes were peeled off from the silicon wafer and cut into rectangular shape with different dimensions. U-shape substrates with different lengths and electrode gaps were 3D printed using rigid resin material (Formlabs Form 3). Then, conductive copper tape was attached on the U-shape substrate as the electrode. Finally, the PDMS membrane was carefully sandwiched between two U-shape substrates with precise control of stretch and then fixed by two long screws. The wind tunnel and cylinders were printed using rigid resin material (Formlabs Form 3) in three parts, which were assembled together to form the complete wind tunnel. An electric fan (SUNON DC 12) was fixed in the rear of the wind tunnel to generate different inlet wind speeds, which can be controlled by adjusting the DC voltage of the fan.

### 4.2 Simulation of the Vortex Street

Simulations of the Kármán vortex street were conducted in Comsol Multiphysics 5.2a. Single-phase laminar flow model was chosen and the medium (air) was set as incompressible. The density and dynamic viscosity of the air were set as 1.184 kg/m^3^ and 1.84 × 10^–5^ Pa·s (25 °C). Except for the inlet and outlet of the channel, all other boundaries were set as a wall without slip. The inlet velocity of the channel was controlled by a piecewise function which could increase the velocity every 5 s and stay at a certain speed of 10 s. The initial velocity and pressure inside the tunnel were set as zero. Finally, a time-dependent solver was chosen to conduct the simulation.

## Supporting Information Available

Photos of the home-designed wind tunnel; illustration of the dimension of the stationary section of the wind tunnel; simulated pressure and velocity contour figures when the *Re* number is the minimum and the maximum; FFT frequency analysis of the simulated vortex street; simulated pressure and velocity of spots at different CLs and at different wind speeds; output short-circuit current of the KVSM-TENG with the vortex street and without the vortex street; transferred charges of the KVSM-TENG with the vortex street and without the vortex street; wind speed calibration of the wind tunnel; vibration frequency of the KVSM-TENG with and without the vortex; simulated pressure and velocity with different cylinder diameters; photo of the KVSM-TENG with the vacuum adsorption effect; simulated pressure and velocity of different heights; outputs of the KVSM-TENG located at different heights; schematic illustration of the vortex flow process when the KVSM-TENG located at different spots; outputs of the KVSM-TENG with different membrane stretches; average power of the KVSM-TENG with and without the vortex street; charging a 47 μF capacitor at the wind speed of 2 m/s; and comparison between electromagnetic, piezoelectric, and triboelectric mechanisms for wind energy harvesting using vortex street (PDF)
Response at cut-in wind speed of 0.52 m/s (MP4)
Vortex shear rate (MP4)
Streamlines (MP4)
Response under gas leakage (MP4)
Pipeline gas leakage alarming (MP4)
