# Supplementary material for: Kármán Vortex Street Driven Membrane Triboelectric Nanogenerator for Enhanced Ultra-Low Speed Wind Energy Harvesting and Active Gas Flow Sensing
Source: ACS Appl Mater Interfaces. 2022 Nov 2;14(45):51018–28. doi: 10.1021/acsami.2c16350 (PMC9673068; doi:10.1021/acsami.2c16350)
Supplement: Supplementary file 1 — am2c16350_si_001.pdf [file am2c16350_si_001.pdf]

Supporting information

Kármán Vortex Street Driven Membrane Triboelectric Nanogenerator for Enhanced Ultra-low Speed Wind Energy Harvesting and Active Gas Flow Sensing

Wenjian Li [1], Liqiang Lu [1], Xianpeng Fu [2], Chi Zhang [2], Katja Loos [3], Yutao Pei [1,*]

[1] Department of Advanced Production Engineering, Engineering and Technology Institute Groningen, Faculty of Science and Engineering, University of Groningen, Nijenborgh 4, 9747 AG Groningen, The Netherlands

[2] CAS Center for Excellence in Nanoscience, Beijing Key Laboratory of Micro-nano Energy and Sensor, Beijing Institute of Nanoenergy and Nanosystems, Chinese Academy of Sciences, Beijing 101400, China

[3] Macromolecular Chemistry and New Polymeric Materials, Zernike Institute for Advanced Materials, Faculty of Science and Engineering, University of Groningen, Nijenborgh 4, 9747 AG Groningen, The Netherlands

* Corresponding author. Email. y.pei@rug.nl

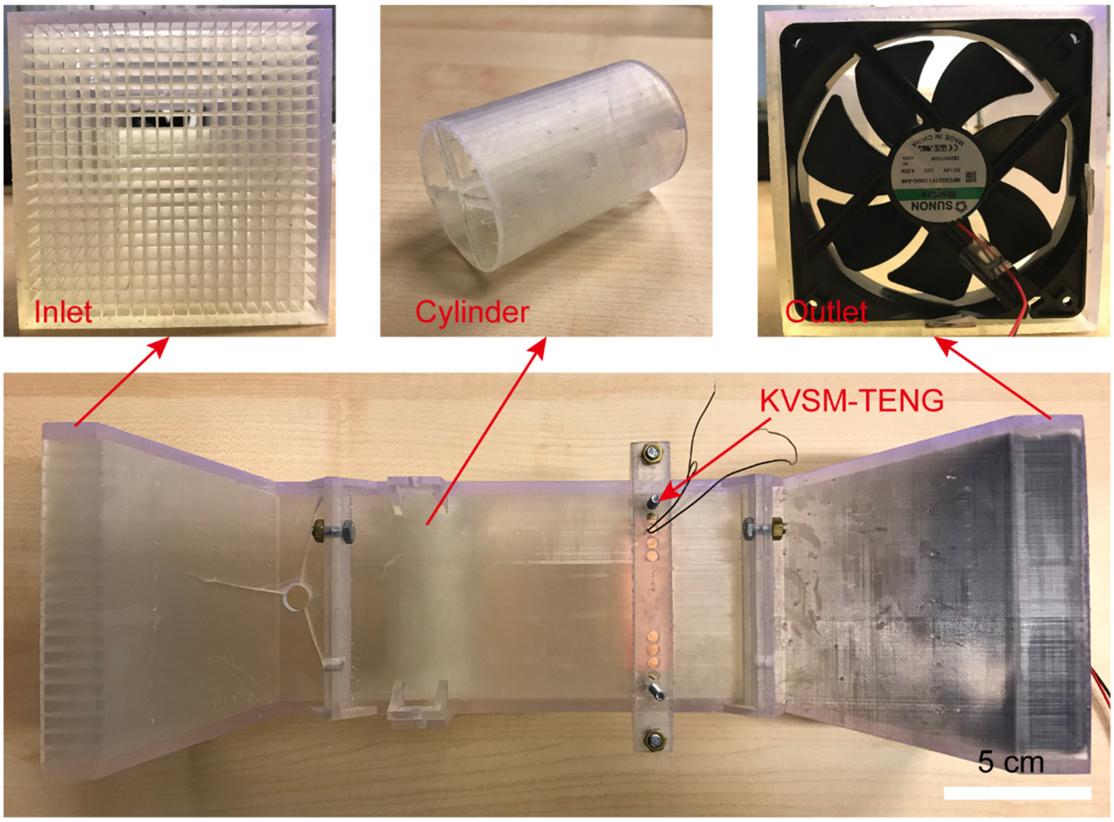

Figure S1. Photos of the home-designed low speed wind tunnel. Scale bar: 5 cm.

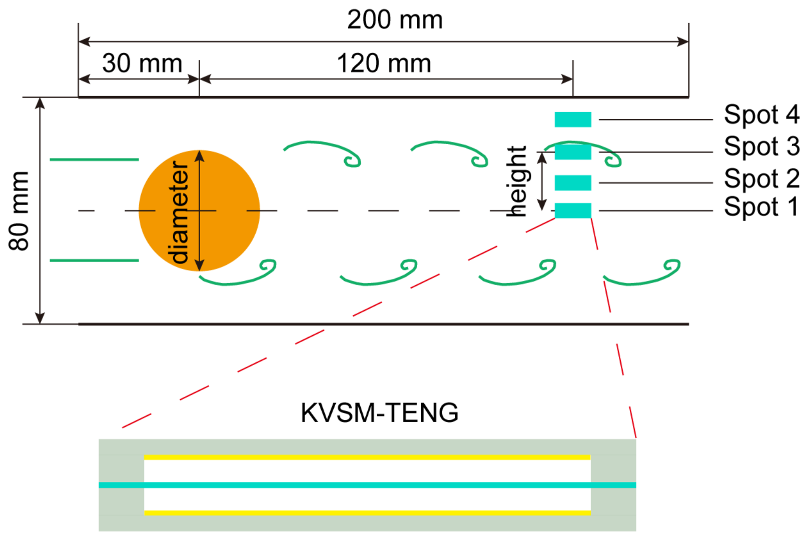

Figure S2. Illustration of the dimension of the stationary section of the wind tunnel as well as the placement location of the cylinder vortex shedder and the KVSM-TENG.

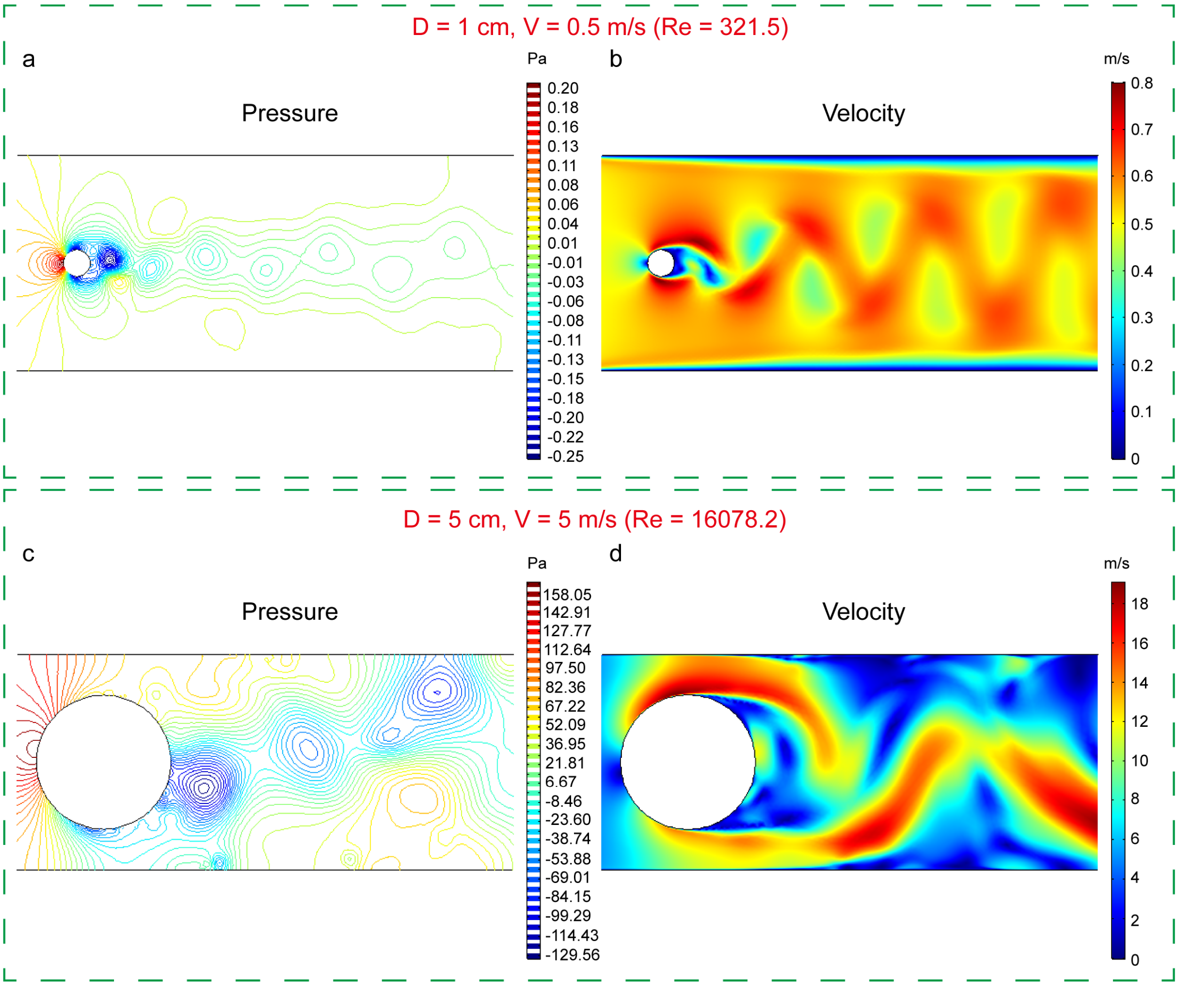

Figure S3. Simulated pressure and velocity contour figures when the *Re* number is the minimum and the maximum. (a) Pressure and (b) velocity contours when the Re number is the minimum (321.5, D = 1 cm, V = 0.5 m/s). (c) Pressure and (d) velocity contours when the *Re* number is the maximum (16078.2, D = 5 cm, V = 5 m/s).

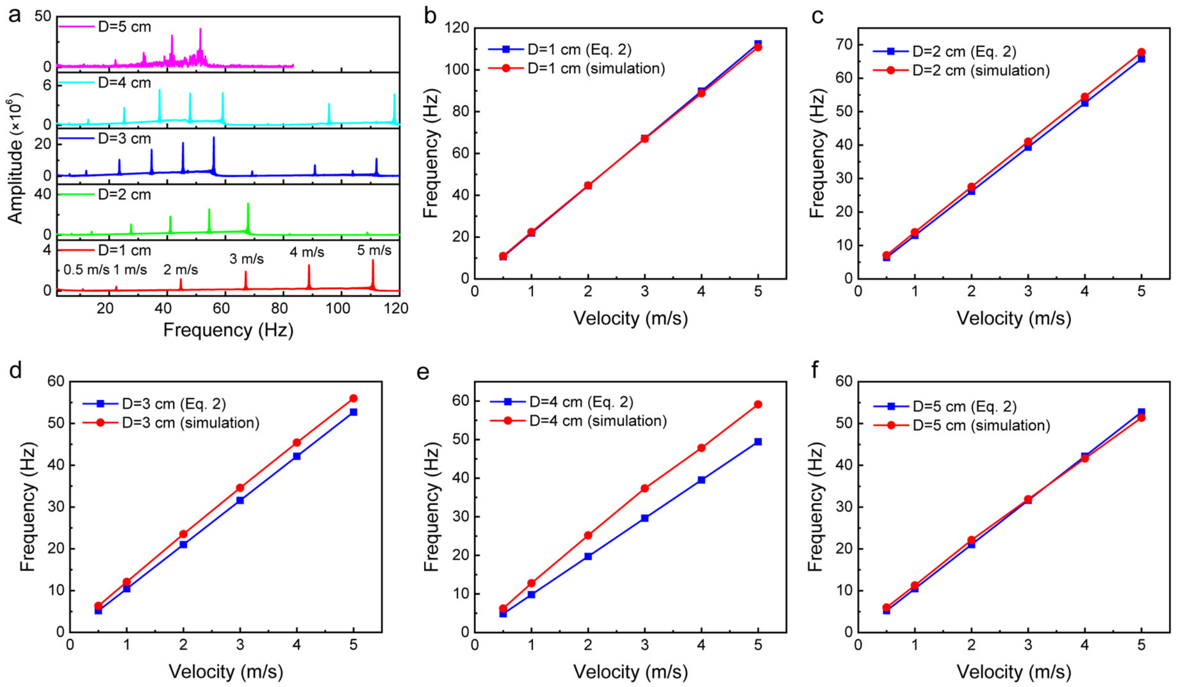

Figure S4. Fast Fourier transform (FFT) frequency analysis of the simulated vortex street. (a) FFT analysis of the pressure contours with different cylinder diameters (1 cm, 2 cm, 3 cm, 4 cm and 5 cm) and inlet wind speeds (0.5 m/s, 1 m/s, 2 m/s, 3 m/s, 4 m/s and 5 m/s). (b-f) Comparison of the simulated frequency and the calculated frequency with Eq. 2 when the diameter is set to 1 cm (b), 2 cm (c), 3 cm (d), 4 cm (e) and 5 cm (f), respectively.

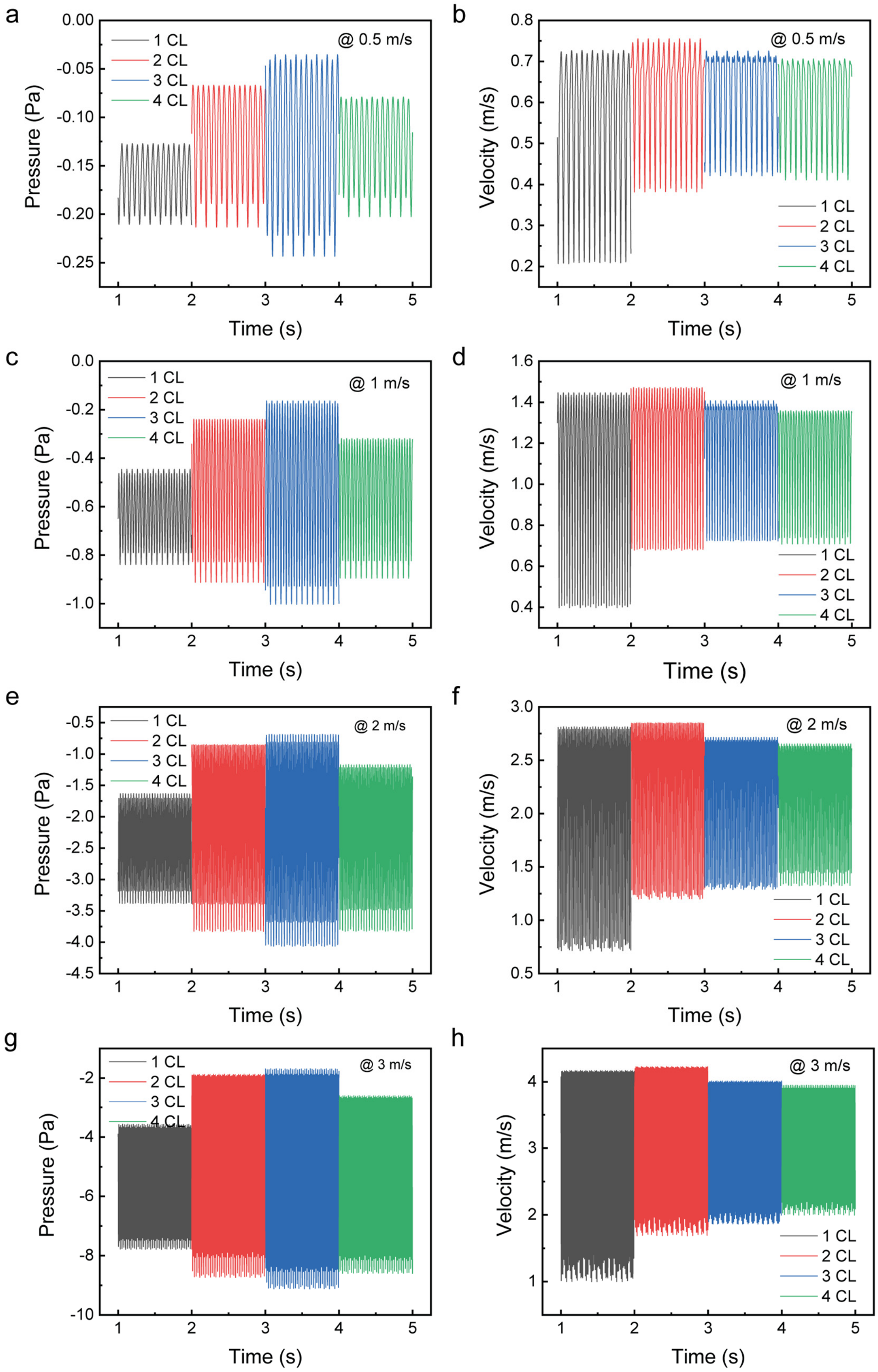

Figure S5. The simulated pressure (left panel) and velocity (right panel) of spots at different characteristic lengths (CL) from the cylinder at different inlet wind speeds (height: 0 cm): (a, b) 0.5 m/s, (c, d) 1 m/s, (e, f) 2 m/s, and (g, h) 3 m/s.

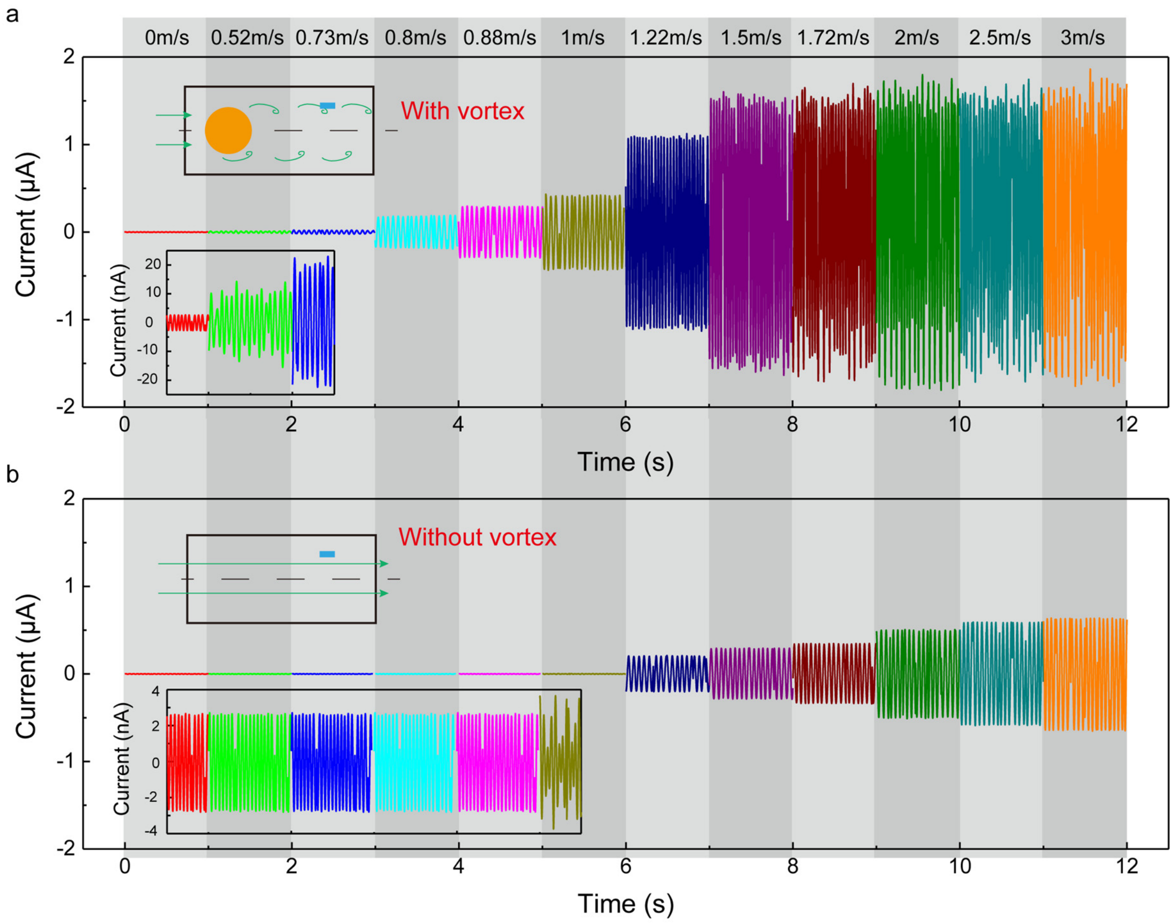

Figure S6. Output short-circuit current of the KVSM-TENG with the vortex street (a) and without the vortex street (b).

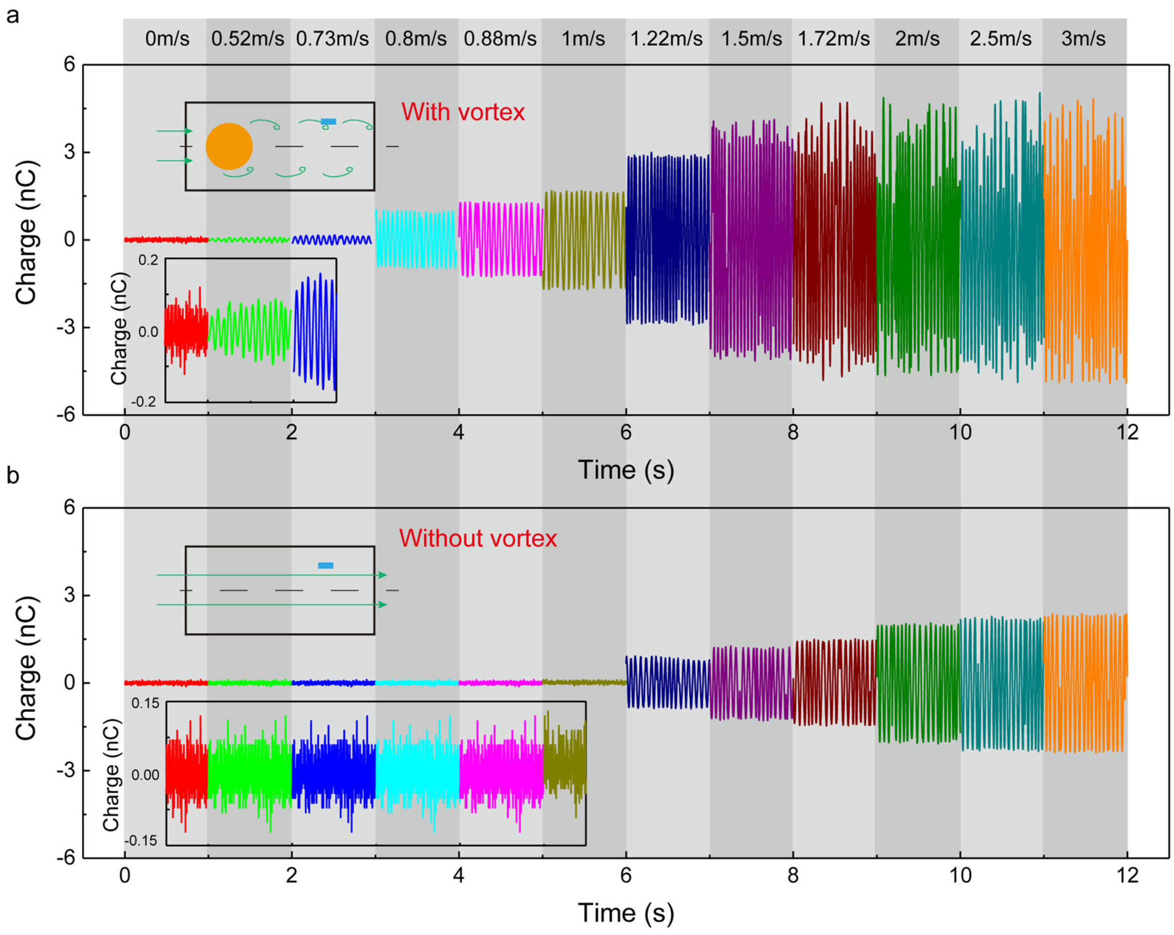

Figure S7. Transferred charges of the KVSM-TENG with the vortex street (a) and without the vortex street (b).

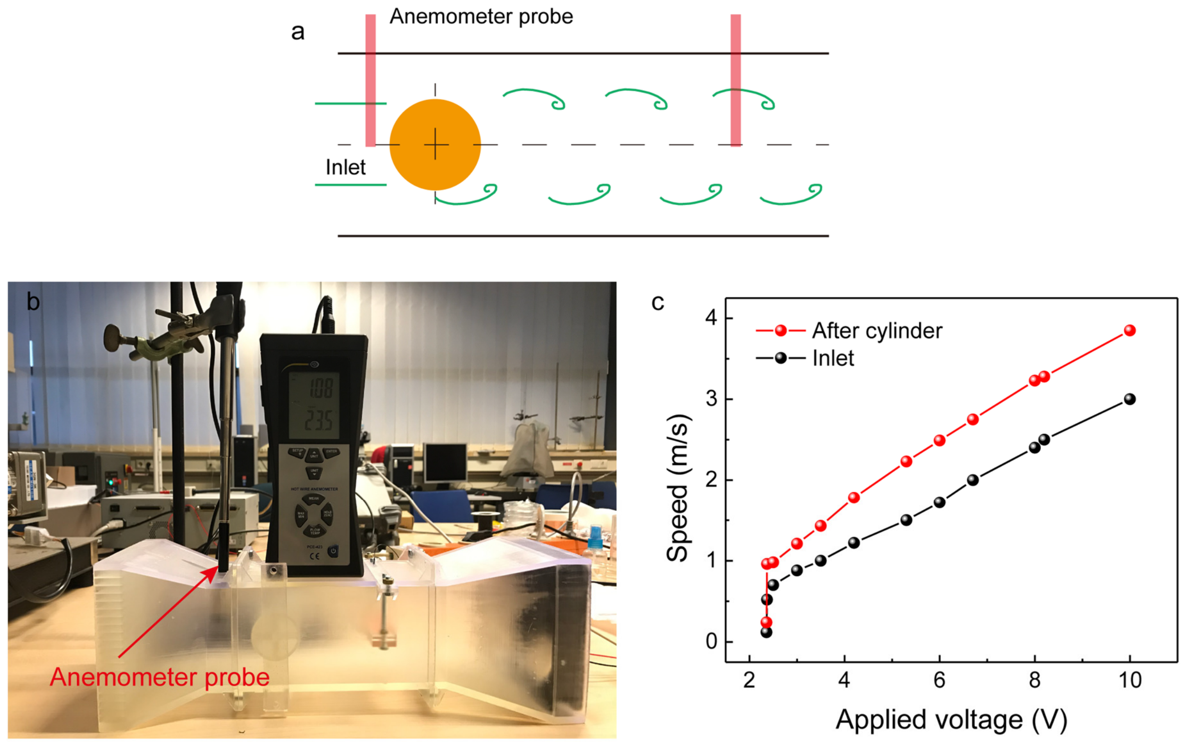

Figure S8. Wind speed calibration of the wind tunnel. a) Schematic illustration of the calibration spots. b) Set-ups of the calibration. c) Calibrated wind speed at the inlet and after the cylinder. The applied voltage refers to the voltage applied to the electric fan to reach different rotation speed.

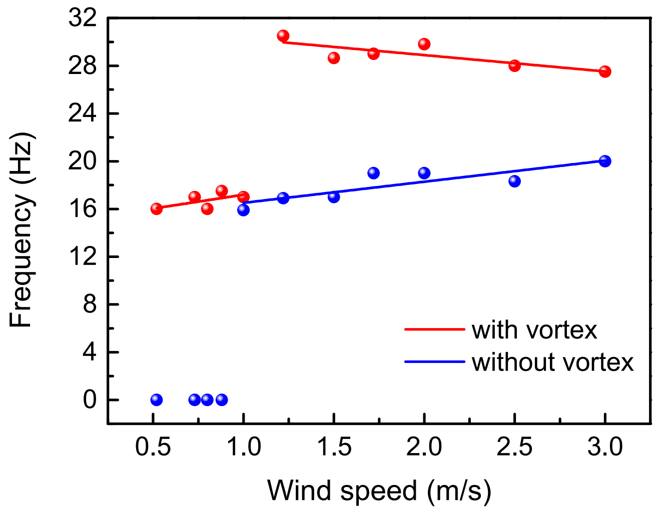

Figure S9. Vibration frequency of the KVSM-TENG with and without the vortex.

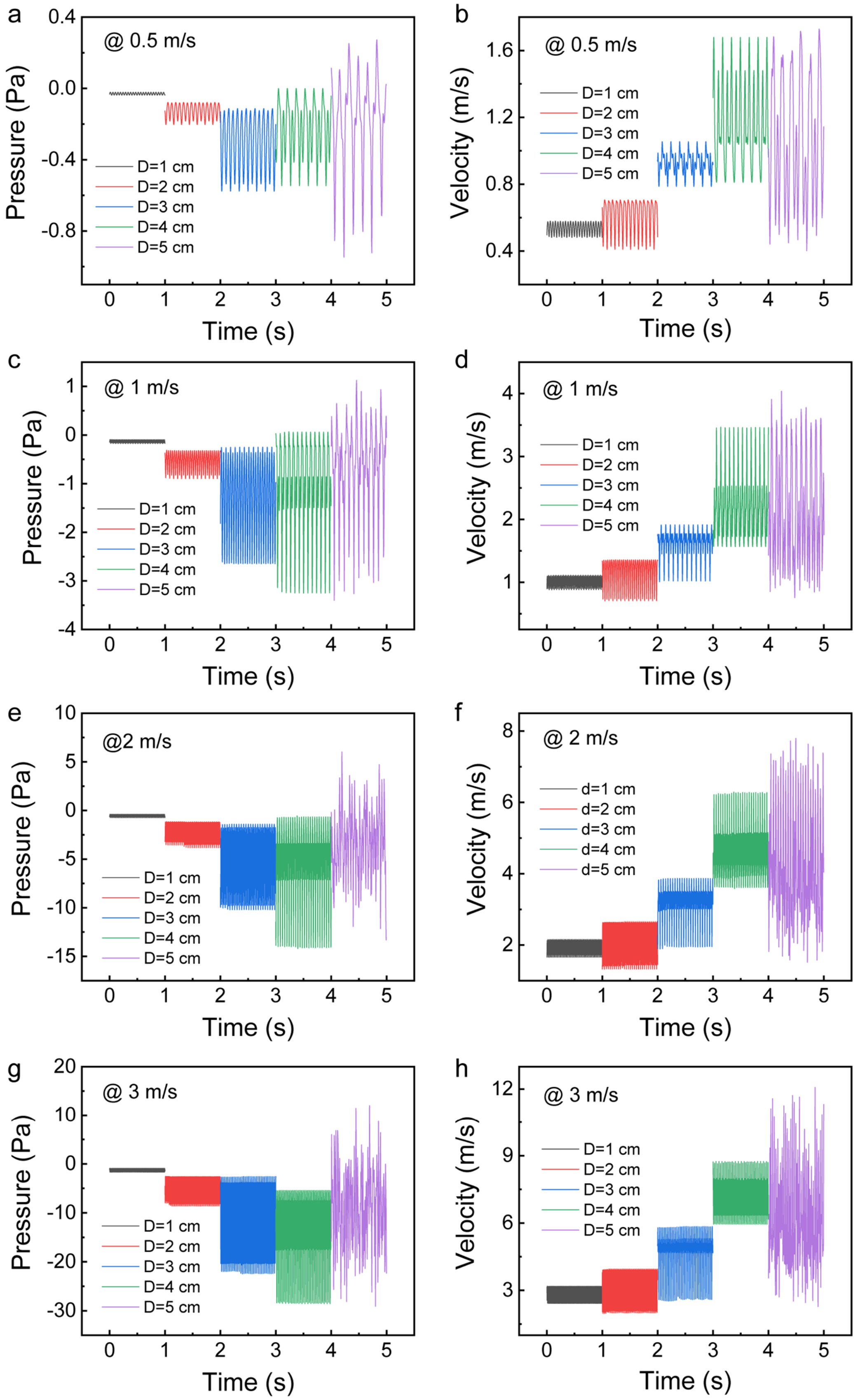

Figure S10. The simulated pressure (left panel) and velocity (right panel) with different cylinder diameters at different inlet wind speeds: (a, b) 0.5 m/s, (c, d) 1 m/s, (e, f) 2 m/s, and (g, h) 3 m/s.

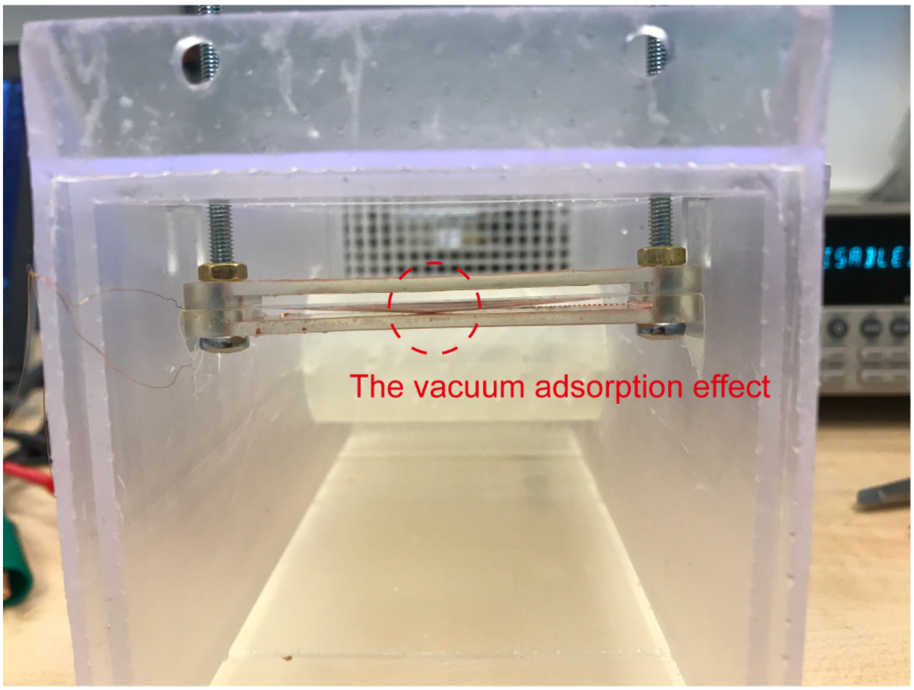

Figure S11. Photo of the KVSM-TENG with the vacuum adsorption effect.

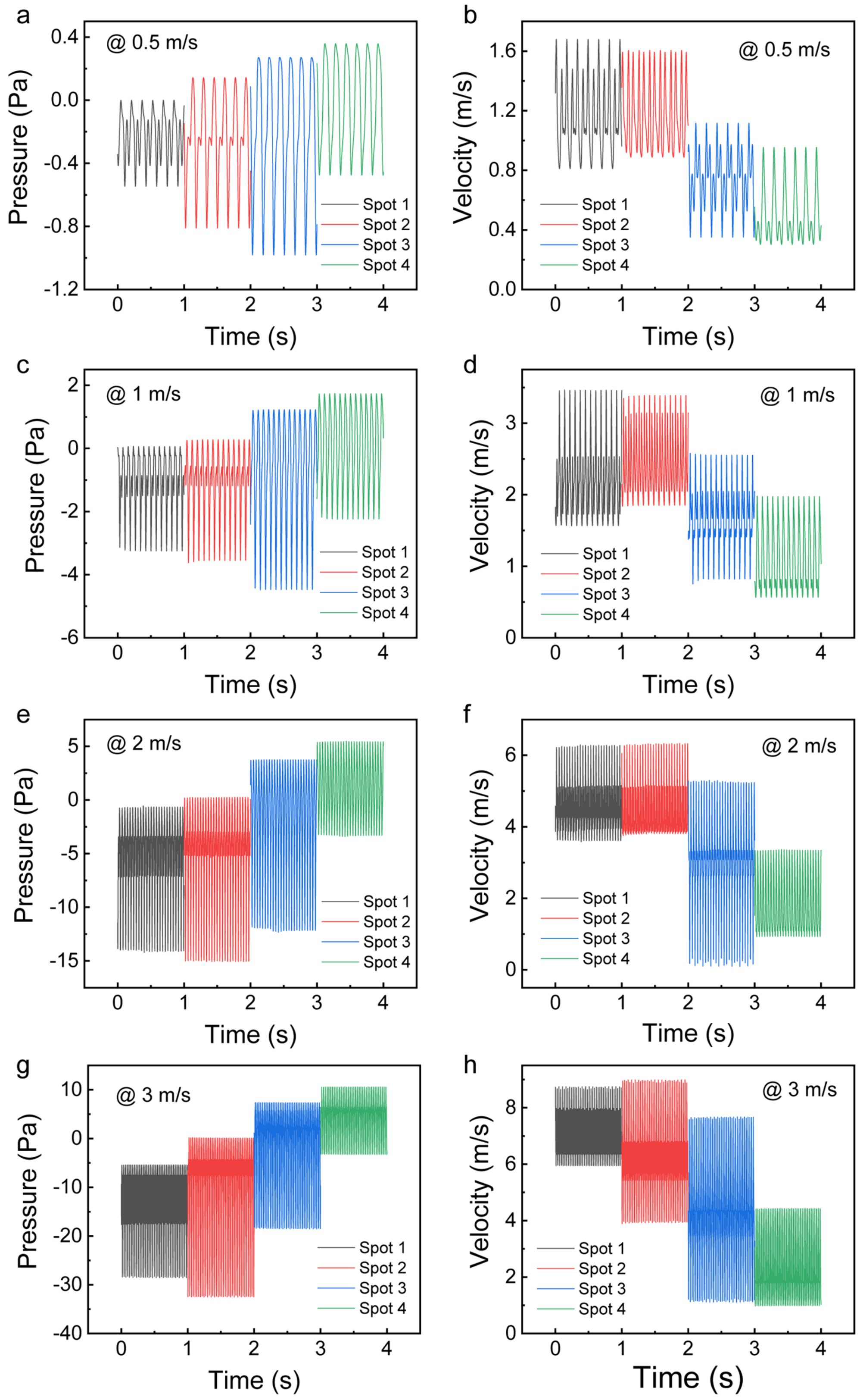

Figure S12. The simulated pressure (left panel) and velocity (right panel) of different heights (spot 1, spot 2, spot 3, spot 4) at different inlet wind speeds: (a, b) 0.5 m/s, (c, d) 1 m/s, (e, f) 2 m/s, and (g, h) 3 m/s.

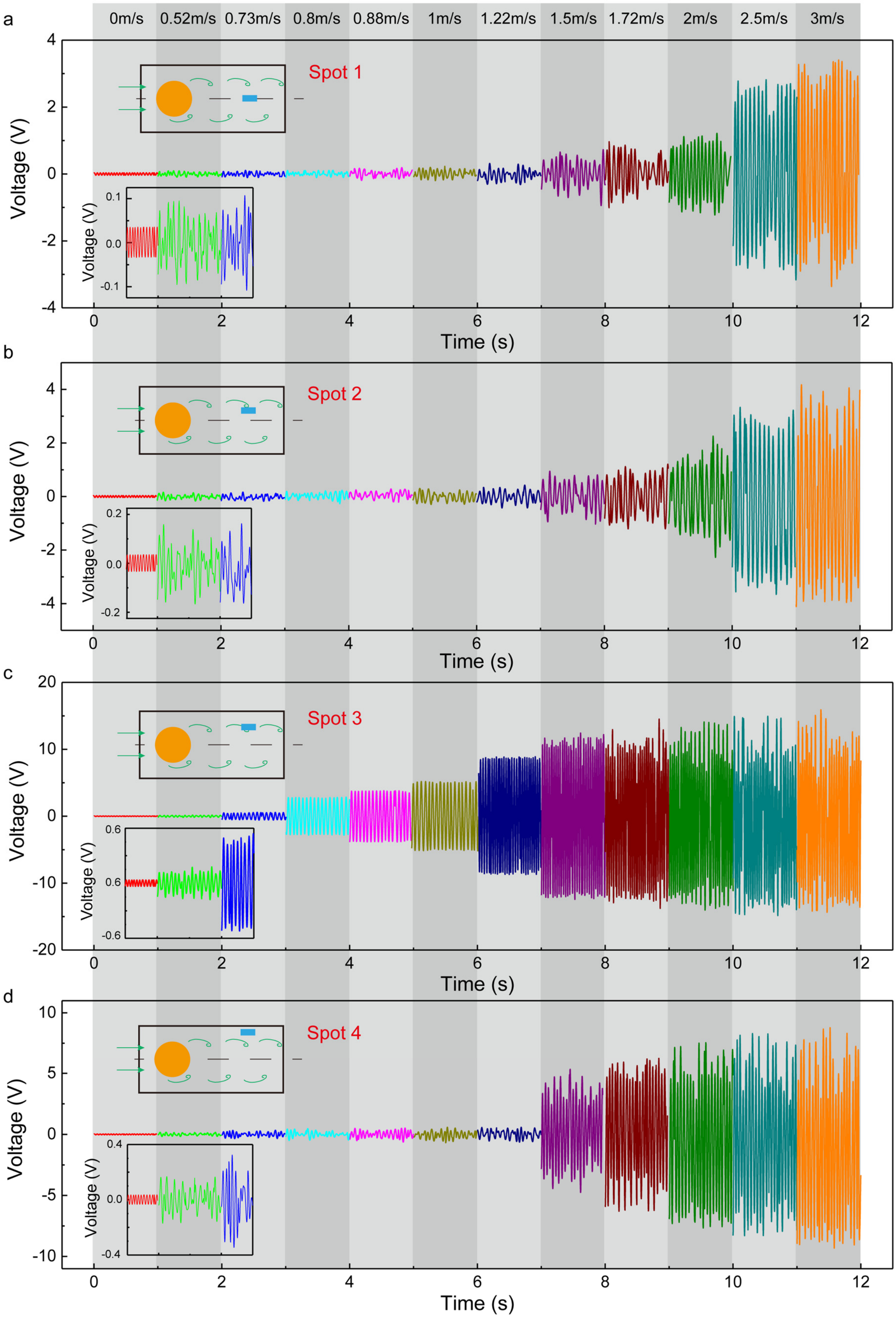

Figure S13. The outputs of the KVSM-TENG located at different heights: (a) spot 1, (b) spot 2, (c) spot 3 and (d) spot 4.

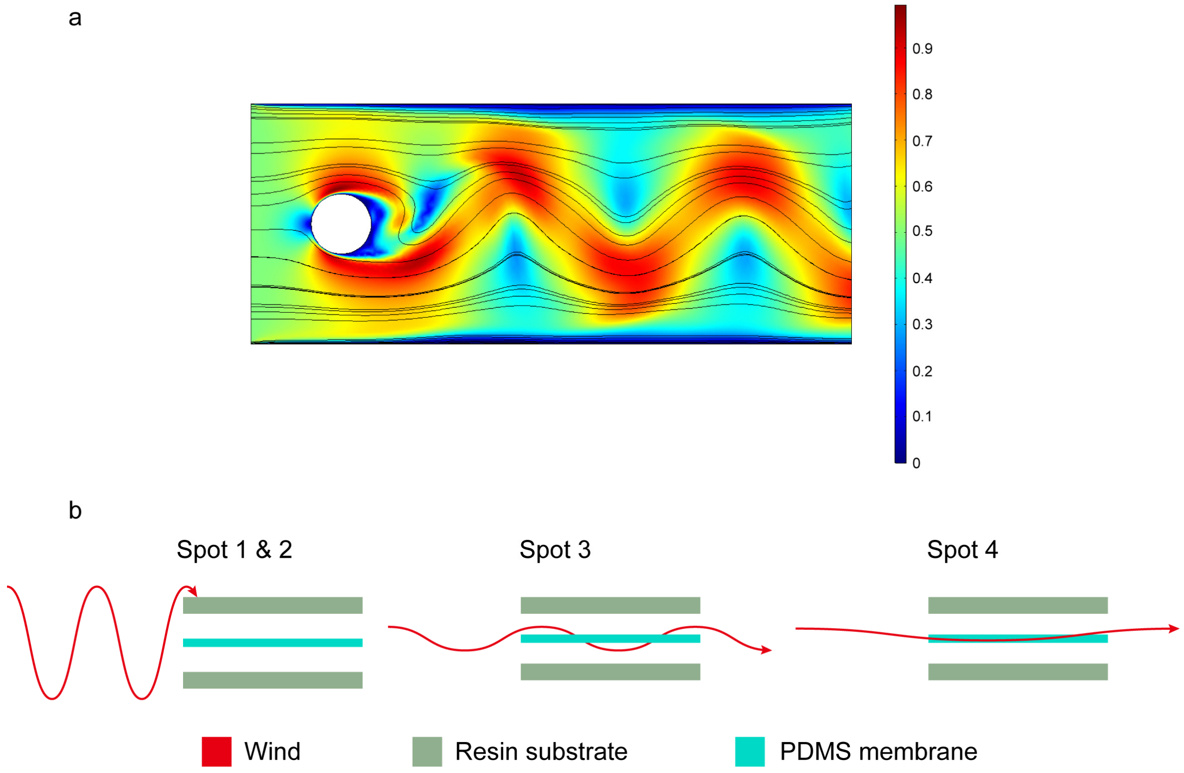

Figure S14. Schematic illustration of the vortex flow process when the KVSM-TENG located at different spots. (a) A velocity contour with streamlines (D = 2 cm, V = 0.5 m/s). (b) Schematic illustration of the fluctuating wind flows across the KVSM-TENG located at different spots.

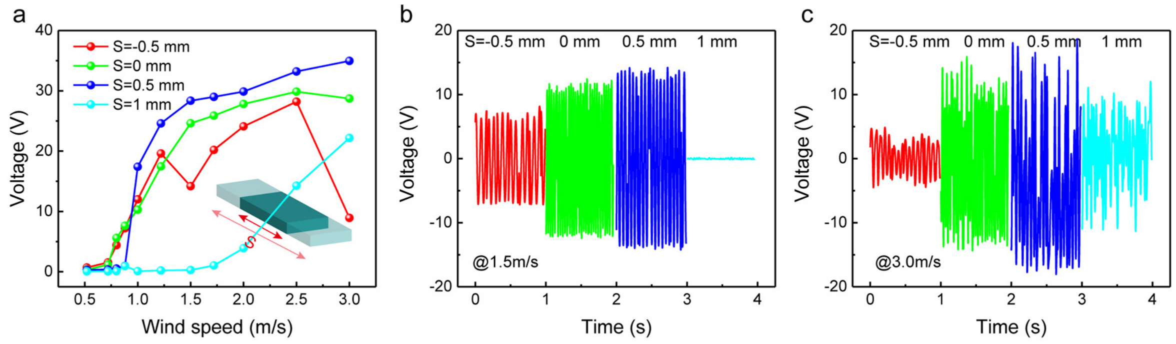

Figure S15. The outputs of the KVSM-TENG with different membrane stretches. (a) The outputs of the KVSM-TENG with different membrane stretches. The outputs of the KVSM-TENG with different membrane stretches at the wind speed of 1.5 m/s (b) and 3 m/s (c), respectively.

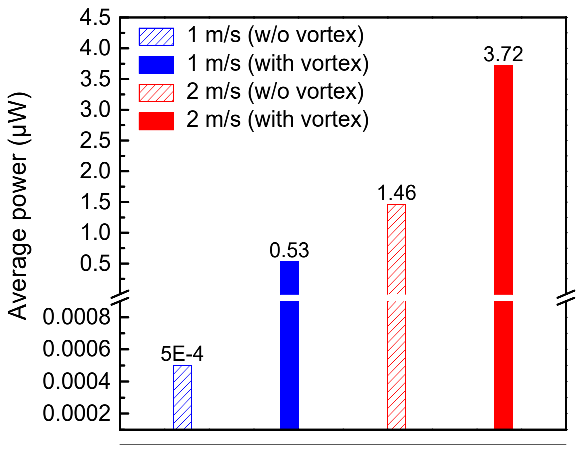

Figure S16. Average power of the KVSM-TENG with and without the vortex street.

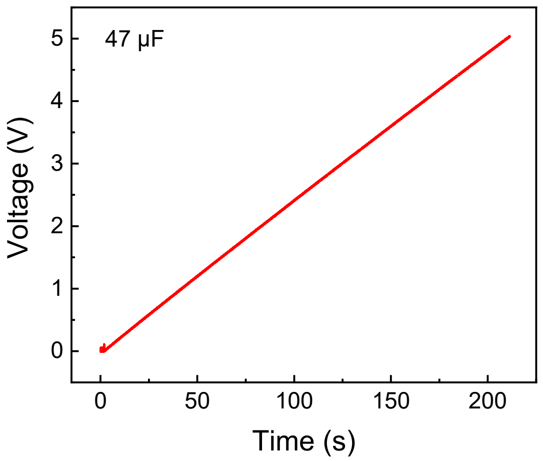

Figure S17. Charging a 47 μF capacitor at the wind speed of 2 m/s.

Table S1. Comparison between electromagnetic, piezoelectric and triboelectric mechanisms for wind energy harvesting using vortex street.

Mechanism	Cut-in wind speed / lowest wind speed applied (m/s)	Output (V)	Power (mW)	References
Electromagnetic	2.3 m/s	-	~0.33 @2.3 m/s	[1]
	3 m/s	6 @ 3 m/s	-	[2]
	1.38 m/s	5.8e-3 @ 3 m/s	1.77e-3 @ 3 m/s	[3]
Piezoelectric	2.3 m/s	10 @ 2.3 m/s	0.1 @ 2.3 m/s	[4]
	0.4 m/s	0.04 @ 0.4 m/s<br>0.1 @ 9 m/s	-<br>-	[5]
	1.25 m/s	0.2 @ 1.25 m/s	-	[6]
Triboelectric	0.52 m/s	10 @ 1 m/s<br>26 @ 2 m/s	0.53e-3 @ 1 m/s<br>3.72e-3 @ 2 m/s	This work

References

[1] Z. Methal, M. S. M. Ali, N. M. Maruai, R. A. Ghani, Microwatt Energy Harvesting by Exploiting Flow-Induced Vibration, J. Adv. Res. Fluid Mech. Thermal Sci. 47 (2018), 25-34.

[2] A. B. Atrah, M. S. Ab-Rahman, H. Salleh, M. Z. Nuawi, M. J. Mohd Nor, N. B. Jamaludin, Karman Vortex Creation Using Cylinder for Flutter Energy Harvester Device, Micromachines (Basel) 8 (2017), 227.

[3] D.-A. Wang, C.-Y. Chiu, H.-T. Pham, Electromagnetic energy harvesting from vibrations induced by Kármán vortex street, Mechatronics 22 (2012), 746-756.

[4] M. Demori, M. Ferrari, A. Bonzanini, P. Poesio, V. Ferrari, Autonomous Sensors Powered by Energy Harvesting from von Karman Vortices in Airflow, Sensors (Basel) 17 (2017), 2100.

[5] S. Petroni, F. Rizzi, F. Guido, A. Cannavale, T. Donateo, F. Ingrosso, V. M. Mastronardi, R. Cingolani, M. De Vittorio, Flexible AlN flags for efficient wind energy harvesting at ultralow cut-in wind speed, RSC Adv. 5 (2015), 14047-14052.

[6] Z. Lai, S. Wang, L. Zhu, G. Zhang, J. Wang, K. Yang, D. Yurchenko, A hybrid piezo-dielectric wind energy harvester for high-performance vortex-induced vibration energy harvesting, Mech. Syst. Sig. Process. 150 (2021), 107212.
